# Extended reality and healthcare practitioner well-being: scoping review

**DOI:** 10.1192/bjo.2025.10858

**Published:** 2025-09-24

**Authors:** Holly Mould, Jonathan R. Abbas, Michael Loizou, Nick Culley, Sheena Asthana, Rohit Shankar, John Downey

**Affiliations:** Aintree University Hospital, NHS University Hospitals of Liverpool Group, Liverpool, UK; ExR Solutions Ltd, London, UK; Centre for Health Technology, University of Plymouth, Plymouth, UK; Plymouth Medical School, University of Plymouth, Plymouth, UK; Cornwall Intellectual Disability Equitable Research, Cornwall Partnership NHS Foundation Trust, Truro, UK

**Keywords:** Extended reality, burnout, workforce, medical technology, scoping review

## Abstract

**Background:**

Extended reality may offer a convenient and effective method of increasing well-being within the wider healthcare workforce and particularly for those working in the mental health sector who are subject to high levels of stress because of increased workload, high levels of staff turnover and limited resources.

**Aims:**

This scoping review aims to identify and assimilate relevant literature pertaining to the use of extended reality to improve healthcare practitioners’ well-being.

**Method:**

Databases (MEDLINE, CINAHL, Cochrane and PubMed) and grey literature were searched for relevant articles using established methodology and reported as per the Preferred Reporting Items for Systematic Reviews and Meta-Analyses (PRISMA) extension for scoping reviews.

**Results:**

A total of 280 articles were yielded by the search strategy, with 13 relevant articles selected by two independent reviewers in a blinded process. Studies demonstrated a heterogenous pool of outcome measurement modalities, intervention modalities and duration and frequency of the interventions. Of all the studies, 85% note a positive impact on healthcare practitioner well-being but studies have limited comparability because of heterogeneity. Interventions were engaging but the practicality of implementing such technologies into a finance- and time-limited healthcare environment will be a challenge.

**Conclusions:**

Whilst extended reality is a promising well-being intervention, there is a paucity of literature relating to its effect on mental health practitioners’ well-being, and further studies in this area are required.

Current staff shortages within the healthcare sector are higher than the national average for other sectors in England.^1^ In particular, there is a shortfall in the capacity of the mental health workforce^1,2^ in a climate of rising numbers of mental health presentations to healthcare providers in the UK.^3^ The mental health workforce experience high levels of work-related stress while having limited resources,^4^ coupled with high levels of staff turnover.^5^ Indeed, high workloads, hindered quality of care, poor commitment to the organisation, lack of investment and feeling unable to contribute to organisational agendas precede staff departures.^6^

Workplace stress and staff burnout negatively affect the health of employees and patient safety, satisfaction and the quality of care.^7^ Mental health workers undertake ‘psychologically hazardous’ work,^8^ and have higher levels of emotional exhaustion than other healthcare practitioners.^4^ The burnout experienced by mental health employees contributes to higher healthcare costs, with one in three mental health nurses experiencing burnout^4^ and staff burnout alone costing the National Health Service (NHS) up to £400 million annually.^9^ Community mental health employees experience greater levels of burnout than other specialities,^4,10^ potentially because of longer hours on shift^11^ or isolated working in the community.^4^

There is a body of evidence that interventions can support the well-being of staff,^12^ but the implementation, heterogeneity, measurement of impact and theoretical underpinning are lacking. Practical, accessible and standardised approaches that can be translated into routine care easily are needed.

Virtual reality can be defined as ‘a three-dimensional computer-generated simulated environment, which attempts to replicate real world or imaginary environments and interactions, thereby supporting work, education, recreation, and health’.^13^ Extended reality may be used as an overarching term to encompass virtual reality, augmented reality and mixed reality.^14^ Augmented reality layers computer-generated objects over real physical objects, allowing the participant to interact with them.^15^ Mixed reality can be thought of more broadly as a blend of the real and virtual worlds.^16^ Virtual reality and extended reality applications are emerging as a potential effective well-being strategy for employees.^17^ Applications of extended reality have proven to be effective in decreasing stress and increasing relaxation in the nascent literature.^18,19^ Virtual reality applications are accessible, cheap and could be integrated into busy environments more easily than other interventions that involve greater resource and organisational burden,^17,20,21^ such as talking therapies or a timetabled well-being programme.

Previous systematic reviews have sought to evaluate the role of virtual reality and extended reality in workplace well-being more widely but have not focused specifically on mental health practitioners.^17,20,21^ This scoping review identifies and analyses the literature relevant to extended reality interventions to improve mental health professionals’ well-being. Because of the emerging nature of the extended reality well-being literature generally, diverse study designs and immature application for healthcare workers, a scoping review was conducted to identify the current knowledge base and map the field.^22^

## Method

A scoping review was performed in five steps, in accordance with the methodology described by Arksey and O’Malley.^23^

### Identifying the research questions

First, the research questions were identified and formulated as follows:

- What peer-reviewed research literature exists that examines the impact of virtual reality and extended reality on healthcare workers?
- Which common themes can be derived from these studies?
- To what extent is this research applicable to mental health practitioners?
- What are the implications of these findings for next steps?

### Identifying the relevant studies

Peer-reviewed articles containing the following terms in their title or abstract were included:

(wellbeing OR well-being OR well being OR quality of life OR wellness OR mental) AND (healthcare workers OR nurs* OR medical workers OR healthcare professionals OR staff) AND (virtual reality OR vr OR augmented reality OR extended reality OR gamification)

Given mixed reality has a broad definition, the authors elected not to include this in the search string because of the risk of inclusion of a wide variety of non-virtual interventions, which would be outside the remit of this scoping review.

Further inclusion criteria were formulated using the population, intervention, comparator and outcomes (PICO) framework,^24^ whereby the population included healthcare professionals, the intervention was the use of extended reality and the outcome was a domain related to well-being, mood or stress. There was no comparator.

Exclusion criteria were articles not written in the English language, opinion pieces, studies in which technology was used for healthcare training purposes or with patients or students in the intervention group.

Articles were identified by searching the literature databases MEDLINE, CINAHL, Cochrane and PubMed. Grey literature was identified by searching these terms on Google Scholar and the King’s fund websites. To ensure that only the most recent advances in technology were included in the search, articles were excluded if they were older than ten years. Grey sources were used to identify peer-reviewed work that may have been missed within core databases, but did not include non-peer-reviewed work.

### Study selection

Duplicates were removed with the use of Mendeley referencing software version 1.19.5 for Windows (Elsevier, London, UK; see https://www.mendeley.com), and the remaining articles underwent blind screening for relevance by two independent reviewers (H.M. and J.D.). Titles and abstracts were pasted into Windows Microsoft 365 Excel spreadsheets and the two reviewers independently gave a yes/no/maybe label and made a memo describing the decision. The respective Excel spreadsheets were then shared to check for agreement. The level of agreement was 82%, with four manuscripts leading to a conflict and three needing a further discussion, because of one reviewer having a ‘maybe’ decision. Conflicts were resolved by meeting to discuss the paper and cross-checking it with the inclusion criteria. Resolution was as followed: three papers were excluded as they were not about extended reality, one was excluded as the cohort was not relevant and one was a protocol design. One conflict was initially included, as upon discussion it met the criteria. A third reviewer was available (M.L.) if a mediator was needed, but this was not the case.

### Charting the data

The lead author developed a data extraction framework and shared it with the team for feedback. A single data extraction process took place whereby prudent information from the included manuscripts were summarised in an Excel spreadsheet. As is suggested from guidance, where one scoping reviewer does the extraction, a proportion (50%) of the outputs were cross-checked by a second reviewer.^25^ Included articles and their contents (authors, location, year, population, methods and sample results) are charted in Table 1.

**Table 1:** Descriptive information of the included articles

Reference	Year	Country	Population	Intervention	Outcome measures	Sample outcomes
Williams & Riches^26^	2023	England	*N* = 14 *F* = 11, *M* = 3 Members of staff working in NHS in-patient psychiatric rehabilitation unit	1 h session of virtual reality relaxation with options of virtual environments	Visual Analogue Scale (VAS)	Participants felt less stressed, anxious and sad, and more relaxed and connected to nature post-intervention.
					Qualitative evaluation of technology	Participants enjoyed the interactive elements of the scenarios and found the virtual reality immersive. Some participants reported unfocused visual elements and stated graphics quality could be improved. Participants were eager to repeat the experience and asked for further sessions.
Hayakawa et al^27^	2022	USA	*N* = 71 Healthcare professionals working in a paediatric hospital	3–5 min sessions of classical music played whilst viewing an alpine scene in virtual reality	Professional Quality of Life	Participants had decreased burnout post-intervention. Participants who completed three virtual reality sessions reported greater levels of compassion satisfaction compared to baseline.
					State-Trait Anxiety Inventory (STAI)	STAI scores decreased post-intervention.
					Caring Ability Inventory	Participants had decreased secondary traumatic stress post-intervention.
Pascual et al^28^	2023	USA	*N* = 32 *F* = 20, *M* = 12 Virtual reality 16, Mobile 16 Members of staff working in an emergency department	Minimum of three sessions of guided meditation within different virtual reality versus mobile environments over 4 weeks	Anxiety Short Form 8a	Improvements in the ‘I am grouchy’, ‘I feel anxious’ and ‘I feel tense’ domains after participants used virtual reality. The other two domains did not show improvement.
					Heart rate variability	Sessions increased heart rate variability, correlating to relaxation, and increased session use increased heart rate variability.
Weitzman et al^21^	2021	USA	*N* = 18 *F* = 5, *M* = 13 Otolaryngology residents	10 min of weekly guided meditation for 16 weeks, delivered by attaching a smartphone to a virtual reality headset	Maslach Burnout Index (MBI)	Participants experienced significantly decreased emotional exhaustion post-intervention
					Subjective enjoyableness, ease of use and utility	Mean enjoyableness was rated as 74/100, ease of learning 65/100 and usefulness as stress management tool 52/100.
Soh et al^29^	2021	Singapore	*N* = 51 (Age *M* 29.82, s.d. 6.70) *F* = 41, *M* = 10 Healthcare professionals	10 min of guided meditation using virtual reality, pre/proceeded by audio-only guided meditation	Depression Anxiety and Stress Scales-21	No significant differences among groups initially. Not repeated post-intervention.
					Practice Quality-Mindfulness	No effect on attention, difficulty in practice or likelihood of engaging in future practice with audio intervention versus virtual reality.
					igroup Presence Questionnaire	Participants felt an increased sense of presence using virtual reality if performed audio practice first.
					Profile of Mood States	Participants reported lower mood disturbance in virtual reality practice if virtual reality performed after audio.
					Qualitative evaluation of technology	Participants noted visual engagement and immersion in virtual reality, which helped their sense of presence; however. some also noted that the graphics were not optimal. Some participants struggled staying awake for whole experience. Participants commented that the headset felt restrictive.
Bodet-Contentin et al^30^	2023	France	*N* = 88 *F* = 71, *M* = 17 Intensive care unit caregivers	8 min virtual reality session of guided breathing exercises	VAS	Participants’ fatigue decreased immediately after the virtual reality session; however, it was the same after the end of the shift regardless of intervention. This suggests virtual reality’s effect is short term. No significant difference in anxiety and satisfaction. Greater feeling of disconnection with the workplace after use of virtual reality.
					MBI	44% of caregivers had low-severity burnout initially.
					Perceived Stress Inventory	No significant difference.
					Qualitative evaluation of technology	22% of virtual reality sessions prompted notification of minor discomfort. for example nausea, dizziness and non-specific discomfort. 82% stated they would be interested in getting a virtual reality system for the unit, and 90% wanted to repeat the experience. 68% of participants were very satisfied or satisfied with the experience.
Gaggioli et al^31^	2014	Italy	*N* = 121 *F* = 73, *M* = 48 Experimental 40 (Age *M* 46.3, s.d. 7.7) Control 42 (Age *M* 42.9, s.d. 10.5) Wait list 39 (Age *M* 39.6, s.d. 9.7) 60 nurses and 61 teachers who had a subjectively high level of perceived stress but low self-efficacy	Two sessions per week for 5 weeks of relaxing and stressful virtual reality scenarios in a cognitive behavioural therapy approach	STAI	Experimental group demonstrated post-intervention reduction in chronic ‘trait’ anxiety.
					Coping Orientation to Problems Experienced	Four subscales improved post-intervention in the experimental group; however, the ‘emotional support skill’ demonstrated significant improvement.
					Perceived Stress Scale	Experimental group demonstrated post-intervention reduction in score.
					Physiological Stress Measure	Experimental group demonstrated post-intervention reduction in score.
					Satisfaction With Life Scale	No significant difference post-intervention.
Adhyaru & Kemp^32^	2022	England	*N* = 39 (Age *M* 36.61, s.d. 10.26) *F* = 32, *M* = 7 Members of staff working in a NHS trauma service	10 min interactive virtual reality relaxation session during breaks	Heart rate	Heart rate reduced over time before, during and after virtual reality.
					VAS	Participants reported increased levels of happiness and relaxation, and lower levels of sadness, anger and anxiety after using virtual reality. No significant difference in levels of anger or vigour after using virtual reality.
					Adaptation of Credibility/Expectancy Rating Scale	Participants reported a medium–high level of satisfaction with virtual reality experience and low levels of aversion.
					Qualitative evaluation of technology	Participants thought positively of the experience and stated it was ‘great’, ‘amazing’ and ‘calming’. Participants wished to include it as part of their workday and use it multiple times a day.
Beverly et al^33^	2022	USA	*N* = 102 *F* = 73, *M* = 28, Other 1 Frontline healthcare professional during COVID-19	3 min 360-degree video of nature scene delivered through virtual reality	VAS	Participants experienced a large reduction in perceived stress after using virtual reality.
Muir et al^34^	2022	USA	*N* = 97 *F* = 85, *M* = 12 *N* = 57 completed the study protocol Members of nursing staff across seven units in a single academic medical centre	2 month use of a relaxation room with well-being resources including a virtual reality headset loaded with 360-degree videos across a variety of categories	Connor–Davidson Resilience Scale	Significant increase in resilience for nurse managers following intervention, and in clinician 3 registered nurses when compared to clinician 1, 2 and 4 registered nurses.
Nijland et al^35^	2021	Netherlands	*N* = 138 *F* = 116, *M* = 22 Virtual reality 86 (Age *M* 42.4, s.d. 12.1) Non-virtual reality 52 (Age *M* 41.7 s.d. 11.5) Intensive care unit nurses during COVID-19	Recommended use of at least 10 min of virtual reality-based relaxation sessions	VAS	Participants experienced a mean decrease of 14% in stress
					Perceived Stress Scale	The population had low perceived stress
					Connor–Davidson Resilience Scale	The population had low stress resilience
Tarrant et al^36^	2022	USA	*N* = 100 *F* = 91, *M* = 9 Audio 50 (Age *M* 40.9, s.d. 13.9) Virtual reality 50 (Age *M* 42.16, s.d. 14.4) Healthcare workers at a single medical centre	5 min 360-degree video viewed in virtual reality versus audio-only with biodata feedback – electroencephalogram (EEG) activity correlated to firefly activity model	Brunel Mood Scale	Virtual reality participants experienced an increase in happiness and calmness post-intervention. Virtual reality participants had a small decrease in confusion, fatigue and vigour. No significant change in anger, tension or depression.
Michael et al^37^	2019	USA	*N* = 27 Nurses working in oncology and substance misuse	6 weeks of headsets with options of eight virtual reality experiences followed by 3 min of guided breathing	Perceived change in mood, utility and benefit	Participants enjoyed and benefitted from the intervention

NHS, National Health Service.

### Collating, summarising and reporting the results

Articles were collated and common themes were identified using content analysis by hand (H.M.). A deductive approach was undertaken where data from prudent mapping domains were used as categories of interest. Descriptive summaries were generated for the included papers and content was packaged together where similar or contrasting data were noted within the included manuscripts.

## Results

**Fig. 1:**
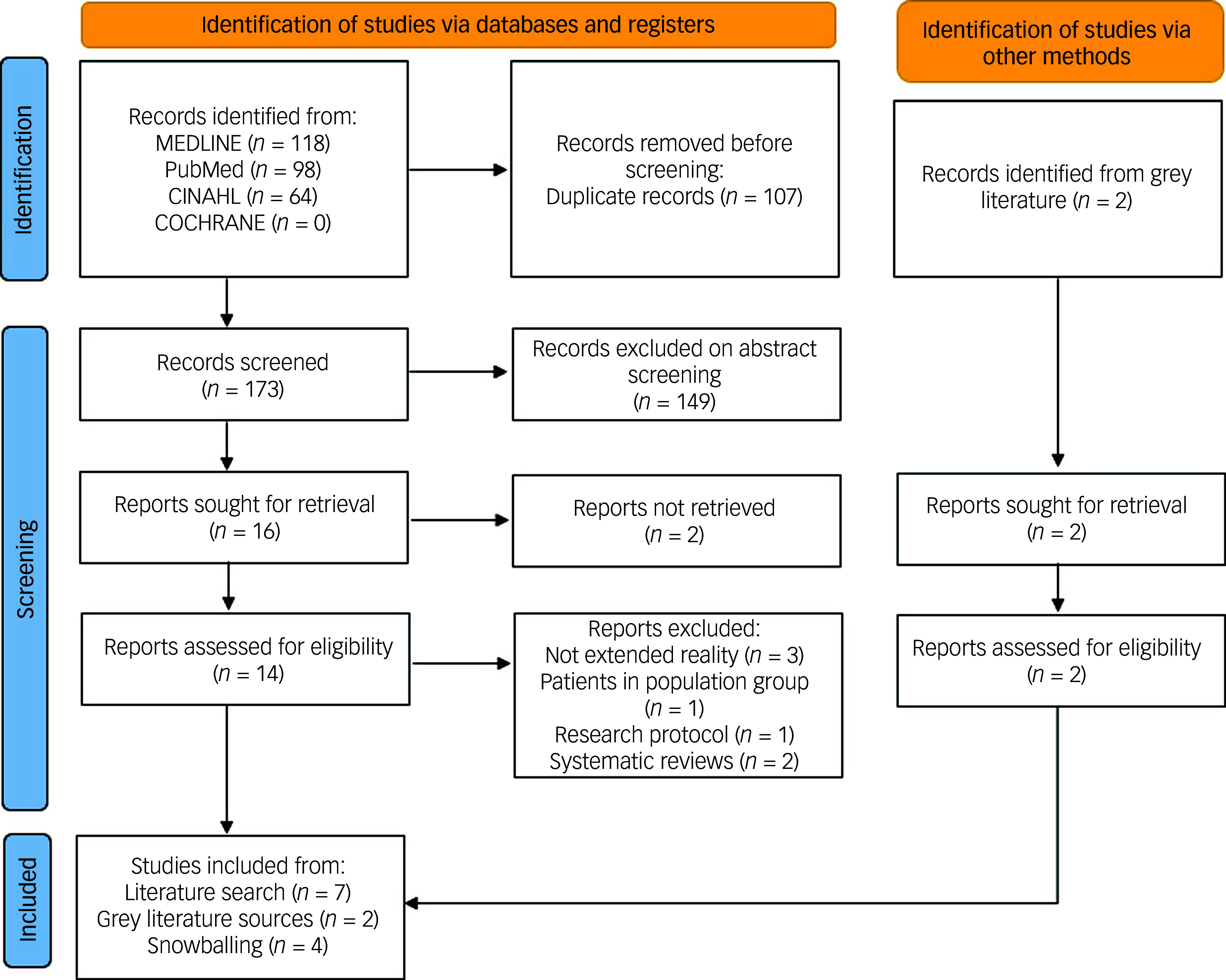
Preferred Reporting Items for Systematic Reviews and Meta-Analyses (PRISMA) flow chart demonstrating the search strategy.

A total of 280 articles were identified using the methodology above (see Fig. 1). As demonstrated in Fig. 1, duplicates (*n* = 107) were removed, and the remaining abstracts (*n* = 173) were screened for relevance. A total of seven relevant articles were identified, with a further two articles found through grey literature searches and four articles from snowballing. Therefore, a total of 13 studies describing primary research met the criteria for inclusion. Studies included healthcare professionals from a variety of countries and were based in a range of settings, with only one being specific to mental health settings.^26^ All studies examined the impact of extended reality interventions on healthcare professionals’ well-being; however, the intervention and outcome measurement modalities differed widely between studies. Two papers were not retrieved, and authors were not contacted as a means to source the papers.

The common themes of these studies included the efficacy, feasibility and acceptability of interventions, alongside the associated financial and logistical considerations that should be considered to increase the engagement and impact of such interventions.

### Intervention modality

Interventions took a variety of forms. Virtual reality was used as a standalone head-mounted device,^26–36^ or with a smartphone placed on to the headset.^21^ Virtual reality interventions included simulations,^26,31,32,37^ 360-degree videos,^27,33,34^ or a combination of the two.^28,35,36^ The 360-degree videos were generally peaceful settings and included footage of beaches, mountains and forests^27,28,33–36^; they were accessed via a specific application^33,35,36^ or through YouTube.^34^ Three studies included virtual reality interventions that had some element of interaction, for example participants being able to plant trees^32^ or pop bubbles.^26,35^ Two studies fed participants’ biodata into the simulation, which altered the appearance of the simulation.^31,36^ Electroencephalogram (EEG) measurements correlating to brain activity were mapped onto a simulated firefly model, which moved below a threshold when the participant was thinking or stressed, and as such reminded participants to attempt to modulate their thoughts.^36^ Another study mapped heart rate to the intensity of the campfire, with the fire eventually extinguishing if the participant was sufficiently relaxed.^31^

Intervention duration varied from 3 min^33^ to at least 10 min and details varied in the hardware used.^21,29,32,35^ Some studies allowed participants to engage with the intervention for as long as they saw fit,^26,34^ whilst some interventions were given as one-off sessions.^26,29,30,33,35,36^ Alternatively, others were delivered at frequent intervals over a longer period of weeks or months.^21,27,28,31,32^ Interventions were delivered in specific sessions,^21,26,29,31,33,35,36^ during normal work breaks,^27,30,32^ or were left in designated spaces for access whenever staff were available.^28,34,37^

### Outcome measurement modalities

**Fig. 2:**
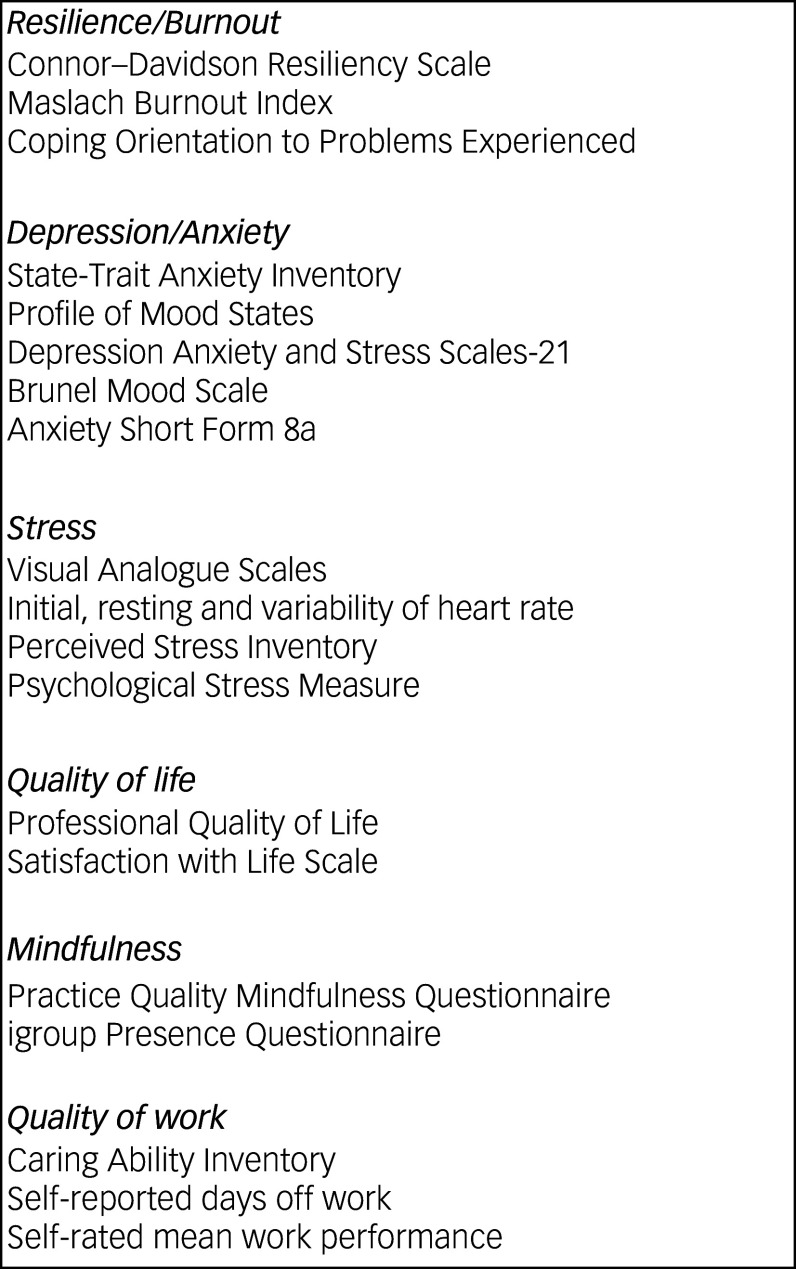
Outcome measures of participant well-being used in the articles.

There are a wide variety of metrics used to measure well-being, stress, resilience, anxiety and depression, as shown in Fig. 2. Domains assessed by studies included resilience, burnout, depression, anxiety, stress, quality of life, mindfulness and quality of work. Metrics ranged from a simple scale where the participant indicated how far along the scale they agree with a sentiment in the Visual Analogue Scale (VAS)^38,39^ to the 22-item Maslach Burnout Inventory.^40^ In most articles, more than one metric was used to give a more rounded sense of participant well-being.

The metrics related to resilience and burnout were as follows: Connor–Davidson Resiliency Scale (measures resilience and ability to cope with stress);^41^ Maslach Burnout Index (assesses burnout levels, concentrating on emotional exhaustion, depersonalisation and personal achievement);^40^ and the Coping Orientation to Problems Experienced (evaluates coping strategies used as a stress response).^42^

Measurements of anxiety and depression utilised in the studies were as follows: the State-Trait Anxiety Inventory (this differentiates between temporary state anxiety and chronic trait anxiety);^43^ Profile of Mood States (assesses anxiety and its impact on daily functioning);^44^ Depression Anxiety and Stress Scales-21 (measures levels of depression, anxiety and stress);^45^ Brunel Mood Scale (evaluates various domains of mood);^46^ and Anxiety Short Form 8a.^47^

Stress measurements used were VASs (linear measures for self-reported stress levels;^38,39^ initial, resting and variability of heart rate variability (a physiological marker of stress);^48,49^ the Perceived Stress Inventory (measures the perception of stress in daily life);^50^ and the Psychological Stress Measure (evaluates the psychological impact of stressors).^51^

Metrics used to assess quality of life were as follows: Professional Quality of Life (ProQOL – measures positive and negative aspects of helping others, including compassion satisfaction and burnout);^52^ and the Satisfaction with Life Scale (assesses overall satisfaction with life).^53^

Quality of work was assessed in some studies using the following metrics: the Caring Ability Inventory (measures the caring ability of individuals in healthcare settings);^54^ self-reported days off work; and self-rated mean work performance (evaluates perceived effectiveness and productivity at work).

Mindfulness was measured by some studies using the following tools: the Practice Quality Mindfulness Questionnaire (evaluates the quality of mindfulness practices); and the igroup Presence Questionnaire (assesses the sense of presence in virtual environments, which can relate to mindfulness).

### Efficacy of interventions

In general, interventions to increase practitioner well-being using extended reality were effective, with 11 out of 13 studies (85%) noting a positive effect on participants. Interventions decreased stress,^26,27,31,33,35^ reduced anxiety^26–28,31,32^ and increased relaxation.^26,31,32^ Metrics relating to job performance were also affected by the interventions, with burnout reduced and compassion increased.^27^ Two studies found that increased use of the intervention correlated with participants feeling more relaxed, with Pascual et al. finding that heart rate variability increased with more virtual reality sessions,^28^ and Gaggioli et al. finding that with increased virtual reality scenario and therapy exposure there was increased relaxation, as assessed by biomarkers.^31^

There were mixed findings relating to fatigue experienced by participants – Soh et al. found that 25% of participants felt fatigued by the virtual reality guided meditation,^29^ whilst Bodet-Contentin et al. and Tarrant et al. found decreased fatigue using their respective metrics^36^ immediately after their virtual reality relaxation sessions.

Interventions were found to be enjoyable^21,32^ and qualitative responses showed the intervention was shown to be ‘great’, ‘amazing’, and ‘really love to be able to do this during my workday’.^32^ Similarly, Weitzman et al. stated that the intervention was very enjoyable (indicated by an average score of 74/100 on the study’s quantitative metric).^21^ Staff said they would recommend interventions to a colleague.^27^ Weitzman et al. and Nijland et al. both found their virtual reality interventions were easy to learn,^21,35^ with those with prior virtual reality experience finding them easier to use.^21^ Interventions that had some elements of interactivity were deemed to be more engaging by participants.^26^

### Feasibility and acceptability

The complexity of the technology may prove to be a barrier in widespread implementation of extended reality-based interventions, as the technological effort required to use the equipment was ‘high’ or ‘very high’. The areas of difficulty reported related to the pairing of a smartphone with biosensors and reading stress data, rather than specifically use of virtual reality hardware.^31^ Williams and Riches found that some technological issues arose during the implementation of the intervention (e.g. freezing of images); however, these were resolved and minimised when a trained facilitator was present.^26^

Few studies commented on the negative aspects of the interventions. Soh et al. found that participants made some comments regarding the use of the hardware, with their virtual reality headset being heavy for participants, and images not being sharp enough.^29^ Bodet-Contentin et al. found that 22% of virtual reality sessions triggered mild side effects, for example nausea and dizziness, but these were not significant enough for participants to terminate the sessions.^30^

Michael et al. provide technical considerations for effective implementation of virtual reality into clinical environments. They note that factors such as maintenance, storage and internet access should all be contemplated before the purchase of hardware and software, and that different headsets and programmes may be more suited to different environments. Indeed, this was the only article to focus on the space requirements for effective virtual reality use, noting that this differs depending on whether the participant is sat or stood. They also state that privacy should be considered, alongside factors that may affect participant comfort, such as ventilation and furniture.^37^

Nijland et al. found that one third of the nurses using their virtual reality intervention felt that they did not have enough time in their working day to use it, and did not feel that they could leave a colleague with their workload to take a break to use the virtual reality.^35^ Similarly, in Michael et al.’s study, whilst all participants felt that they had support from a senior colleague to engage with virtual reality well-being activities, they still felt that they would struggle to integrate the interventions into scheduled breaks.^37^

### Costs of hardware and software

Weitzman et al. note that virtual reality is ‘cheap and accessible’ technology,^21^ but as with many of the studies included in this review, do not state the costs of their software and hardware. Gaggioli et al. commented on the costs of using virtual reality rather than traditional cognitive behavioural therapy to aid well-being; however, they also noted that there was an 85% decrease in costs of one piece of equipment from the initiation to the end of the study because of rapid technological advancement.^31^ Hayakawa et al. state their study equipment was donated, but hardware costs ranged between USD $600 and USD $1300.^27^

## Discussion

There is limited literature pertaining to the use of extended reality to improve healthcare worker well-being, with only one study examining the use of virtual reality to improve mental health staff well-being.^26^ Given the wide variety of technological interventions and metrics used in the literature, the results of studies are difficult to compare. There is a need for further research to be done in this field, given the challenges within the mental health workforce. Although this scoping review has included a heterogenous pool of studies, all were healthcare professionals that work within a stressful and busy clinical environment and, therefore, sentiments of what has been found in this review may be portable to mental health practitioners.

### Effects of interventions

Extended reality interventions have a positive effect on healthcare staff well-being, regardless of whether they are used as short one-off interventions or in longer term programmes. Multiple metrics were used to assess well-being, but studies demonstrate that extended reality can reduce stress, increase relaxation and reduce anxiety. This correlates with previous studies demonstrating that pleasant and immersive virtual environments decrease stress and increase relaxation within the general population and those with mental health conditions.^19,55,56^ Many interventions were one-off interventions with few studies demonstrating the long-term follow-up impact of the interventions. However, as suggested in previous systematic reviews,^57,58^ future research should explore ideal duration and frequency of interventions, with long-term effect on well-being also assessed.

There was no consensus on the appropriate length or frequency of well-being interventions, with interventions ranging from 3 min to 12 week programmes. Some studies opted to leave the intervention in situ for many weeks or months, with staff able to access them as desired. Interventions that have scheduled sessions for staff should ensure that each member of staff has equal access, and that their access can be planned for in terms of staffing levels. In addition, these sessions can be supervised by a technician, and as appropriate may include a debrief. Unsupervised sessions increase the availability of such interventions but rely on staff members to take control of their own well-being and find time to undertake such activities, with some indicating a need for accessibility outside traditional working hours.^37^

It must be questioned as to whether some of these measurement modalities are appropriate as a surrogate measure for well-being. Well-being is a complex concept with multiple modalities and measuring one aspect does not confer one’s well-being. For example, heart rate variability may confer stress in the moment but not overall practitioner well-being, and evaluation tools for depression and anxiety may aid in giving a sense of a practitioner’s psychological well-being, but will may not reflect their well-being as a whole.^59^ A more homogenised measure of well-being is required, which will also aid comparison between the efficacy of interventions in future studies.

### Engagement with interventions

Generally, extended reality is viewed as an engaging and novel activity in the broader literature,^60,61^ and similarly the healthcare professionals in the studies reviewed indicated participants would recommend extended reality to colleagues,^21,27^ suggesting they would like to engage with well-being activities as part of their normal working day.^32^ Staff generally engaged well with such well-being interventions, and they are viewed as enjoyable and beneficial.

As with previous research,^62^ although participants recognise the need for well-being activities, they struggle to find time in the working day to complete them. Even when the intervention was scheduled into rotas with appropriate cross-cover of colleagues, there was still guilt experienced by staff undertaking well-being activities.^35,37^ To combat this, some studies supplied the intervention for use during rostered breaks, which increased relaxation and happiness and decreasing anxiety,^27,32^ but limited the time that the intervention could be used for. Indeed, a previous systematic review of well-being strategies for mental health practitioners found that well-being interventions were difficult to implement effectively into scheduled breaks, most often because of poor staffing levels.^63^

### Feasibility

Although the systematic review by Riches et al. pertains to workplace well-being generally,^17^ rather than specifically a healthcare setting, heaviness of the headset was noted in one study, as was cybersickness in another. Issues with hardware and software are still of note and should be considered when implementing such technology into the clinical environment. Worryingly, a third of doctors state that they do not have the necessary technology to perform their job without disruption – which includes WiFi and broadband.^64^ Reliable internet access may therefore prove to be an issue in integrating extended reality software into the clinical environment, and this should be considered when choosing appropriate applications for well-being activities. Applications that can be downloaded and stored may be better suited to the clinical environment than software that requires an active internet connection for streaming content such as videos.

Financial cost of new equipment will be a barrier to integration of new technologies within the NHS. Ways of making interventions cheaper have been demonstrated in some studies, including the use of the participant’s own smartphone with headsets rather than the use of virtual reality head-mounted devices.^29,37^ There was no consensus among the included articles on the financial viability of extended reality-based interventions. Although initial investment costs for hardware are quoted as being high,^27^ balanced with the cost of burnout and work hours lost, effective well-being interventions in the form of extended reality are likely to be cost-effective. For instance, the cost of paying one nurse a day of sick leave because of stress or burnout (£162.84)^65^ and hiring an agency nurse to cover their shift (£305.88)^66^ is nearly £500 for a single 12 h shift, which is more than the cost of most virtual reality headsets on the market currently (£289.99 for Meta Quest 3S at the time of writing).^67^ Indeed, extended reality is now more affordable,^18^ even more so on an organisational rather than commercial level. However, there are additional costs to consider when running extended reality interventions, such as the purchase of software, maintenance and cleaning costs and the provision of adequate space in which to run interventions. None of the articles evaluated cost-effectiveness of virtual reality as a well-being intervention. Given that many of these costs are ‘one-off’ software and hardware costs, extended reality-based well-being interventions may prove to be cost-effective, but further research is required to evaluate this fully.

### Limitations

Few of the studies included examined long-term effects and outcomes of the technologies, which is common in similar research, and experimental research with robust designs is lacking.^18^ Two papers were not retrieved, and only English manuscripts were considered, creating a blind spot within the scoping exercise. In addition, the aim was to map the current literature specifically to the mental health workforce; however, there is a paucity of extended reality well-being research in this setting, limiting transferable learning. There was limited literature pertaining to extended reality well-being interventions other than virtual reality, and thus conclusions regarding virtual reality technologies may not be applicable to wider extended reality technologies.

### Implications for practice

Extended reality interventions offer a potential way of increasing well-being within the healthcare workforce, as showcased in this scoping review, reported in line with guidance.^68^ They may be cost-effective but require careful consideration of individual software, space requirements and implementation issues, and greater empirical testing is needed to demonstrate the return on investment. A consensus on ideal intervention time and frequency to have the maximal impact on staff well-being is required and homogeneity of outcome measurement modalities needs to be increased in future studies to compare their impact with the current literature.

## Data Availability

No primary data was collected; however, the Excel spreadsheets used to screen and synthesise the literature are available upon request from the corresponding author.
